# Transcriptomic profiling identifies differentially expressed genes associated with programmed cell death of nucellar cells in *Ginkgo biloba* L.

**DOI:** 10.1186/s12870-019-1671-8

**Published:** 2019-02-28

**Authors:** Dahui Li, Di Wu, Shizhou Li, Ning Guo, Junshan Gao, Xu Sun, Yongping Cai

**Affiliations:** College of Life Science, Anhui Agricultural University, Hefei, 230036 China

**Keywords:** *Ginkgo biloba* L., Nucellus, Ovule, Pollen chamber, Programmed cell death (PCD), Transcriptomics

## Abstract

**Background:**

Previously, we demonstrated that pollen chamber formation (PCF) in *G. biloba* ovules was a process of programmed cell death (PCD) within the nucellar cells at the micropylar end. However, the signal triggering the cascades of the programmed events in these nucellar cells remains unexplored.

**Results:**

A transcriptomic strategy was employed to unravel the mechanism underlying the nucellar PCD via the comparative profiles of RNA-seq between pre-PCF and post-PCF ovules. A total of 5599 differentially expressed genes (DEGs) with significance was identified from *G. biloba* ovules and classified into three main categories of GO annotation, including 17 biological processes, 15 cellular components and 17 molecular functions. KEGG analysis showed that 72 DEGs were enriched in “Plant hormone signal transduction”. Furthermore, 99 DEGs were found to be associated with the PCD process, including the genes involved in ethylene signaling pathway, PCD initiation, and PCD execution. Moreover, calcium-cytochemical localization indicated that calcium could play a role in regulating PCD events within the nucellar cells during pollen chamber formation in *G. biloba* ovules.

**Conclusions:**

A putative working model, consisting of three overlapping processes, is proposed for the nucellar PCD: at the stage of PCD preparation, ethylene signaling pathway is activated for transcriptional regulation of the downstream targets; subsequently, at the stage of PCD initiation, the upregulated expression of several transcription factors, i.e., *NAC*, *bHLH*, *MADS-box*, and *MYB*, further promotes the corresponding transcript levels of *CYTOCHROME C* and *CALMODULIN*s, thereby, leads to the PCD initiation via the calcium-dependent signaling cascade; finally, at the stage of PCD execution, some proteases like metacaspases and vacuolar processing enzyme for hydrolysis, together with the process of autophagy, play roles in the clearance of cellular components. Afterwards, a pollen chamber is generated from the removal of specific nucellar cells in the developing ovule.

**Electronic supplementary material:**

The online version of this article (10.1186/s12870-019-1671-8) contains supplementary material, which is available to authorized users.

## Background

During the reproductive development of *Ginkgo biloba* L., pollen chamber functions as a storage site for immature pollens pollinated onto the ovule [1]. Pollen chamber formation (PCF) is resulted from the degeneration of 5 ~ 7 layers of nucellar cells at the micropylar end of ovule [2]. Previous researches have demonstrated that the nucellar degeneration should involve programmed cell death (PCD), due to the occurrence of molecular and biochemical markers for PCD, including DNA ladder and terminal deoxynucleotidyl transferase-mediated dUTP nick end labeling (TUNEL) positive labeling on these nucellar cells, together with morphological characteristics, for instance, nuclear degradation, vacuole rupture, and the process of autophagy [2–4].

PCD represents a common mechanism underlying various developmental processes in both animals and plants [5–7]. In plants, developmental PCD (dPCD) has occurred concomitantly with reproductive and vegetative developments, for instance, cell death of the nucellar tissue, tapetum, sex determination, endosperm, embryonic suspensor, xylogenesis, organ senescence, and aerenchyma formation [8–10]. Other types of PCD have also been found to occur during hypersensitive response against invading pathogens [11], and in response to various abiotic stresses [12, 13].

Unlike the animal PCD, i.e., typically apoptosis which is under control by homologs of Bcl-2 proteins or caspases, the plant PCD is executed in a distinctive, plant-specific way, due to the lack of homologs of apoptotic regulators and executors [14]. A certain degree of common in the regulatory network of the diverse cases, which should be coordinated with the PCD preparation, initiation and execution, has been documented in the modulation of plant dPCD [8, 10]. Similar to many plant developmental processes, dPCD is frequently coordinated by hormone signaling through transcriptional control, most commonly ethylene [8, 15–17]. Ethylene and its signaling pathways promotes several types of dPCD, such as root aerenchyma formation and leaf senescence [17–19]. For other phytohormones, gibberellic acid (GA), abscisic acid (ABA), brassinosteroid (BR), jasmonic acid (JA), and salicylic acid (SA) are found to be regulators of leaf senescence-associated PCD [20]. Additionally, GA could promote tapetum PCD [21], and auxin, cytokinin and brassinosteroid promote the PCD during tracheary element (TE) differentiation [22].

Downstream of the hormone signaling, PCD initiation of particular cell types is triggered by diverse events like finetuning of cytoplasmic [Ca^2+^], cytoplasmic acidification, ROS accumulation, and cytoskeleton modification, respectively or redundantly to a certain extend [8, 13, 23, 24]. Several hydrolytic enzymes have been isolated as putative PCD executers including nucleases and various proteases. For instance, the nuclease BFN1and the aspartate protease PASPA3 from the xylem undergoing PCD, played roles in DNA fragmentation and cellular corpse clearance [23, 25, 26]. The cysteine protease CEP1 functions as an executor during Arabidopsis tapetal PCD [27], as well as XCP1 and XCP2 during TE PCD [28]. Another PCD executors are metacaspases, a type of arginine/lysinespecific cysteine proteases distantly related to animal caspases [29]. A type II metacaspase (McII-Pa) is required for suspensor PCD [30]. The metacaspases MC1 and MC9 are involved in pathogen-triggered PCD and TE PCD, respectively [31, 32]. Other plant proteases, including vacuolar processing enzymes (VPEs), are also shown to be frequently involved in plant PCD. In these cases, nucellain, a barley homologue of VPE, is involved in nucellar PCD during the developing barley grain [33], and δVPE is responsible for the PCD in Arabidopsis seed coats [34]. And the aspartic proteases OsAP25 and OsAP27 could promote tapetum dPCD in rice [35].

In the present study, we conducted a transcriptomic analysis to compare RNA-seq profiles between the ovules before PCF (i.e., pre-PCF) and those after PCF (post-PCF). The aims of this study are (i) to investigate patterns of enrichment for Gene Ontology (GO) annotation and Kyoto Encyclopedia of Genes and Genomes (KEGG) pathways associated with the significantly differentially expressed genes (DEGs); (ii) to elucidate modulating pathways with respective to the nucellar PCD during pollen chamber formation in *G. biloba* ovules. Our results from GO and KEGG enrichments of DEGs between pre-PCF and post-PCF ovules presented possible candidates involved in the nucellar PCD and allowed us to identify components within regulatory and signaling pathways. These transcriptomic data will be useful for determining the molecular mechanism by which pollen chamber is formed in *G. biloba* ovules.

## Results

### Pollen chamber formation within *G. biloba* Ovule

**Fig. 1:**
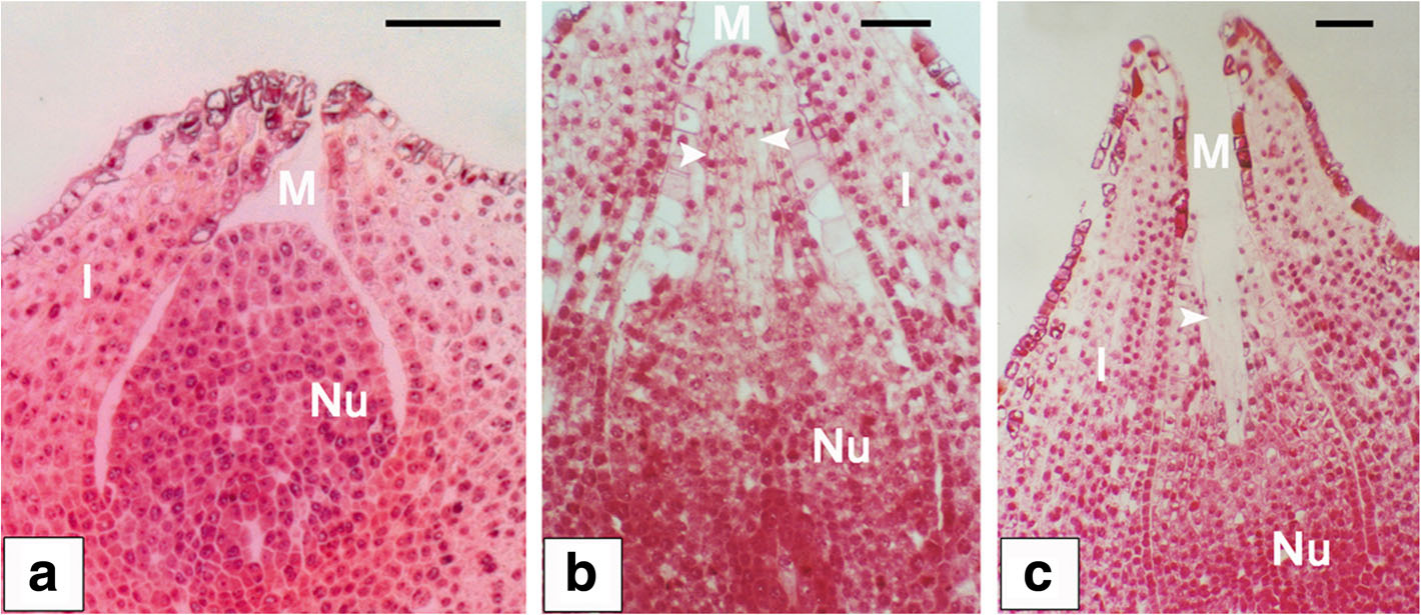
Pollen chamber formation within *G. biloba* ovule observed under the microscope. (**a**) At the early stage of ovule development, nucellar cells are morphologically similar in their size and shape. (**b**) At the later developmental stage, nucellar cells at the micropylar end (indicated by arrows), are elongated longitudinally, and distinguishable from other nucellar cells. (**c**) With the developmental process, pollen chamber (indicated by an arrow) is formed after the death and clearance of nucellar cells at the micropylar end within an ovule. Bars = 250 μm. Abbreviations: I, integument; M, micropyle; Nu, nucellus

During the process of pollen chamber formation within *G. biloba* ovule, several stepwise developmental phases in morphology have been observed (Fig. 1). At the early stage of ovule development, nucellar cells at the micropylar end are morphologically similar in their size and shape with those at other parts of nucellus (Fig. 1a). At the later developmental stage, three to four layers of nucellar cells at the micropylar end are differentiated into longitudinally elongated-shape (Fig. 1b). Finally, these elongated nucellar cells are dead and removed for the formation of pollen chamber within an ovule (Fig. 1c).

**Table 1:** Summary of read numbers based on the RNA-Seq data from *G. biloba* ovules

Sample	pre-PCF	post-PCF
Raw reads	22,246,632 (100%)	22,374,995 (100%)
Total base pairs (Mb)	50.99	50.97
Clean reads	22,216,542 (100%)	22,347,120 (1000%)
Total mapped reads	20,203,385 (90.94%)	20,430,564 (91.42%)
Unique match reads	19,666,682 (88.52%)	19,776,546 (88.50%)

### Sequencing data analysis

In the present study, two transcript libraries from *G. biloba* ovules at the stages of pre- and post- PCF were assayed by high throughout RNA-seq, resulting in 22,246,632 and 22,374,995 raw reads, respectively (Table 1). With the process of quality control for raw reads, clean reads were collected from the two libraries and mapped to the *G. biloba* genome, producing 90.94% (20,203,385 reads) and 91.42% (20,430,564 reads) matched reads in pre-PCF and post-PCF libraries, respectively. Further analysis revealed that 19,666,682 reads (88.52%) in pre-PCF library and 19,776,546 reads (88.50%) in post-PCF library were uniquely matched (Table 1).

**Fig. 2:**
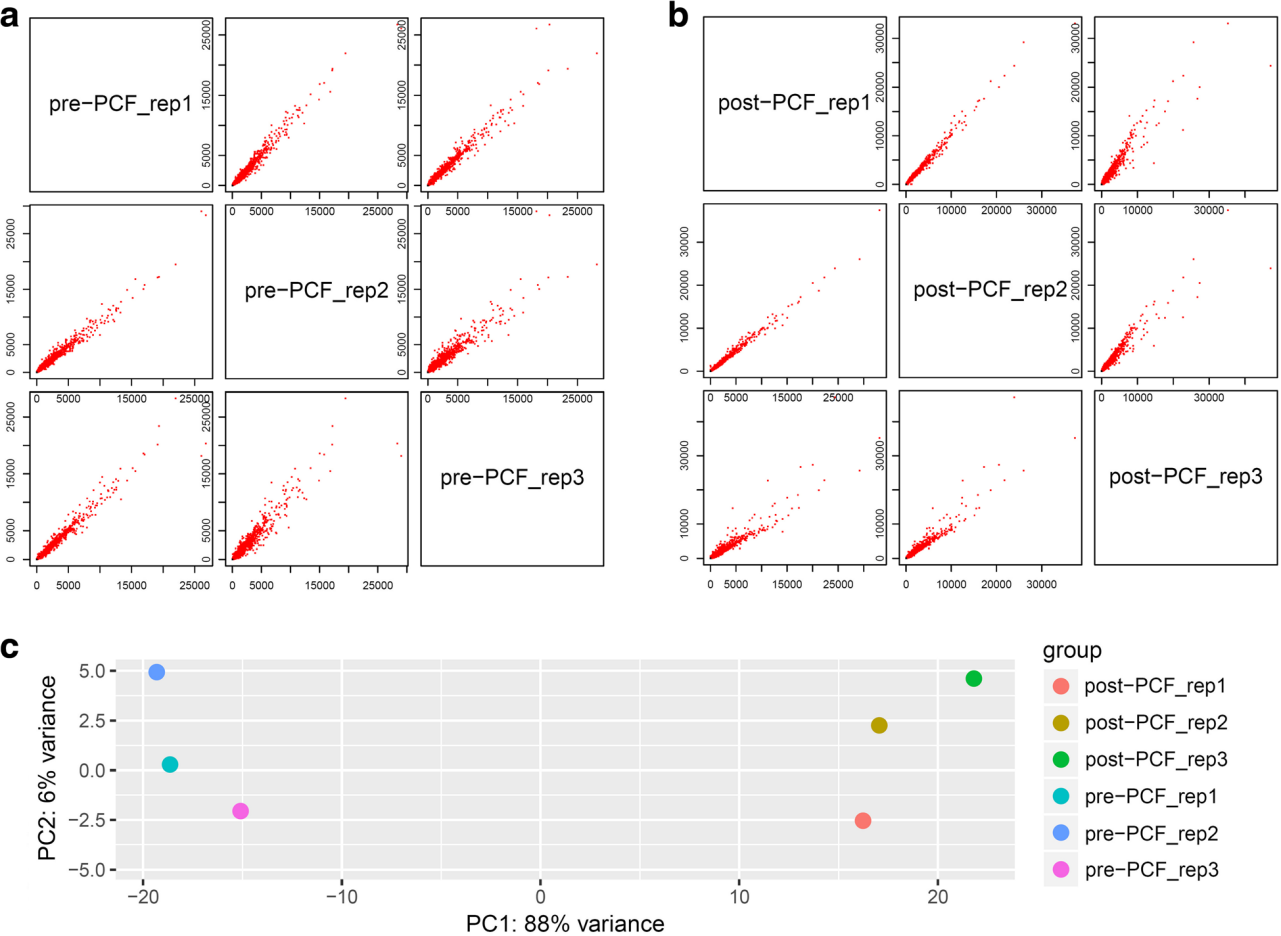
The assessment of correlations among the tested *G. biloba* ovules by analysis of replicate scatter (**a** and **b**) and PCA (**c**), with each sample group (pre-PCF or post-PCF) including three biological replicates (rep_1, 2, and 3), respectively

To assess the reliability of the tested samples, the varied degrees among intra- or inter-groups were performed with analysis of principal component (PCA) and replicate scatter (Fig. 2). Both read-counts from the intra-replicates of pre-PCF and post-PCF showed linear-type scatter and correlations, respectively (Fig. 2a and b). On the contrary, there were significant difference between inter-groups (pre-PCF vs. post-PCF) with 88% variance, compared to 6% variance within the intra-groups (Fig. 2c). These results suggested that sampling of *G. biloba* ovules in the present experiment were reliable and suitable for further analysis.

### Identification and functional enrichment analysis of DEGs

**Fig. 3:**
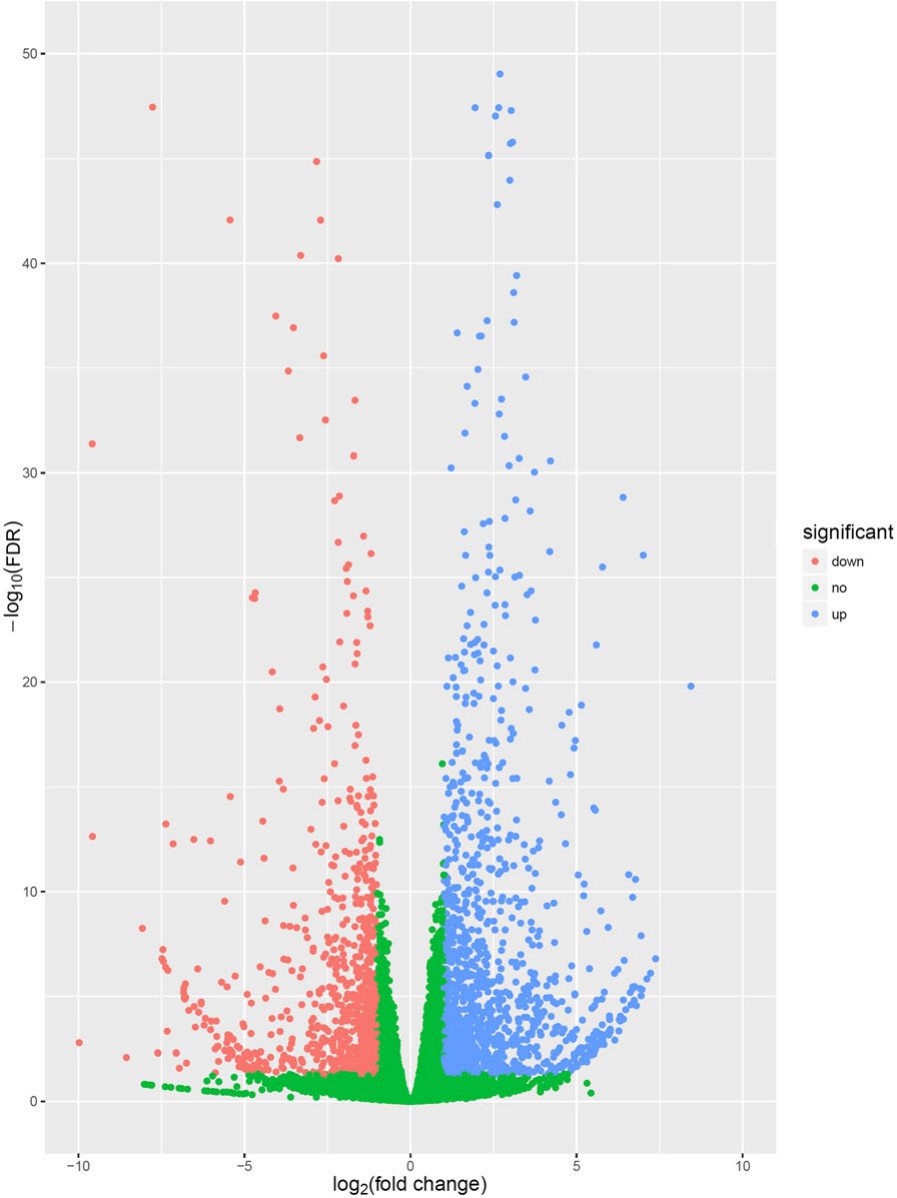
The expression profiles of the identified DEGs. Red and blue points represent the significant DEGs with *FDR* ≤ 0.05 and log_2_(fold change) > 1, and green ones show those without significance, respectively. Fold change refers to the values of FPKM change of post-PCF vs. pre-PCF libraries

Based on the FPKM method, the transcript abundance of each gene from pre-PCF and post-PCF data was analyzed. A total of 21,435 DEGs were identified between pre-PCF and post-PCF libraries (Fig. 3 and Additional file 1: Table S1). Out of these DEGs, 5599 genes exhibited a significant difference in their expression levels with the threshold of *FDR* ≤ 0.05, including 2533 up-regulated and 3066 down-regulated ones (Additional file 1: Table S1).

**Fig. 4:**
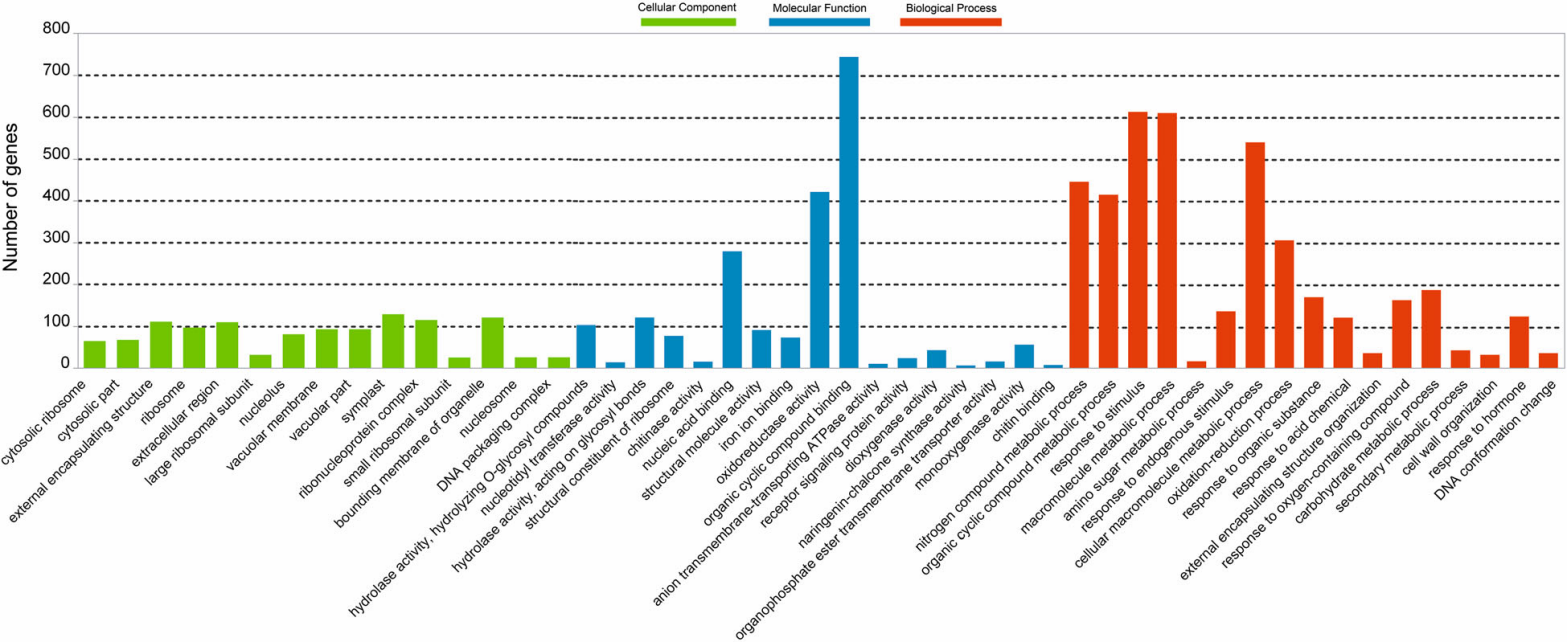
The Gene Ontology (GO) classification of 5599 DEGs. GO terms are summarized in three main categories of cellular component, molecular function and biological process

These identified DEGs were annotated with 17 biological processes, 15 cellular components and 17 molecular functions in GO categories (Fig. 4), and significantly enriched (*P* ≤ 0.001) into 106 GO terms (Additional file 2: Table S2). The terms of “binding” (GO:0005488), “organic cyclic compound binding” (GO:0097159), “heterocyclic compound binding” (GO:1901363), “oxidoreductase activity” (GO:0016491), “nucleic acid binding” (GO:0003676), and “hydrolase activity” (GO:0016798 and GO:0004553) were the dominant groups in the molecular functions; “primary metabolic process” (GO:0044238), “response to stimulus” (GO:0050896), “macromolecule metabolic process” (GO:0043170), “nitrogen compound metabolic process” (GO:0006807), “response to chemical” (GO:0042221), and “oxidation-reduction process” (GO:0055114) were of the representative groups in the biological processes. Among the cellular components, a great number of DEGs were focused on categories of “symplast” (GO:0055044), “cell-cell junction” (GO:0005911), “plasmodesma” (GO:0009506), “bounding membrane of organelle” (GO:0098588), “ribonucleoprotein complex” (GO:0030529), “external encapsulating structure” (GO:0030312), “cell wall” (GO:0005618), and “extracellular region” (GO:0005576).

Moreover, GO terms associated with PCD or autophagy, were found among some of the DEGs, for instance, “programmed cell death” (GO:0012501), “induction of programmed cell death” (GO:0012502), “regulation of programmed cell death” (GO:0043067), “positive of regulation of programmed cell death” (GO:0043068), “negative regulation of programmed cell death” (GO:0043069), “singlet oxygen-mediated programmed cell death” (GO:0010343), “autophagy” (GO:0006914), “process utilizing autophagic mechanism” (GO:0061919), and “ribonuclease activity” (GO:0004540).

**Fig. 5:**
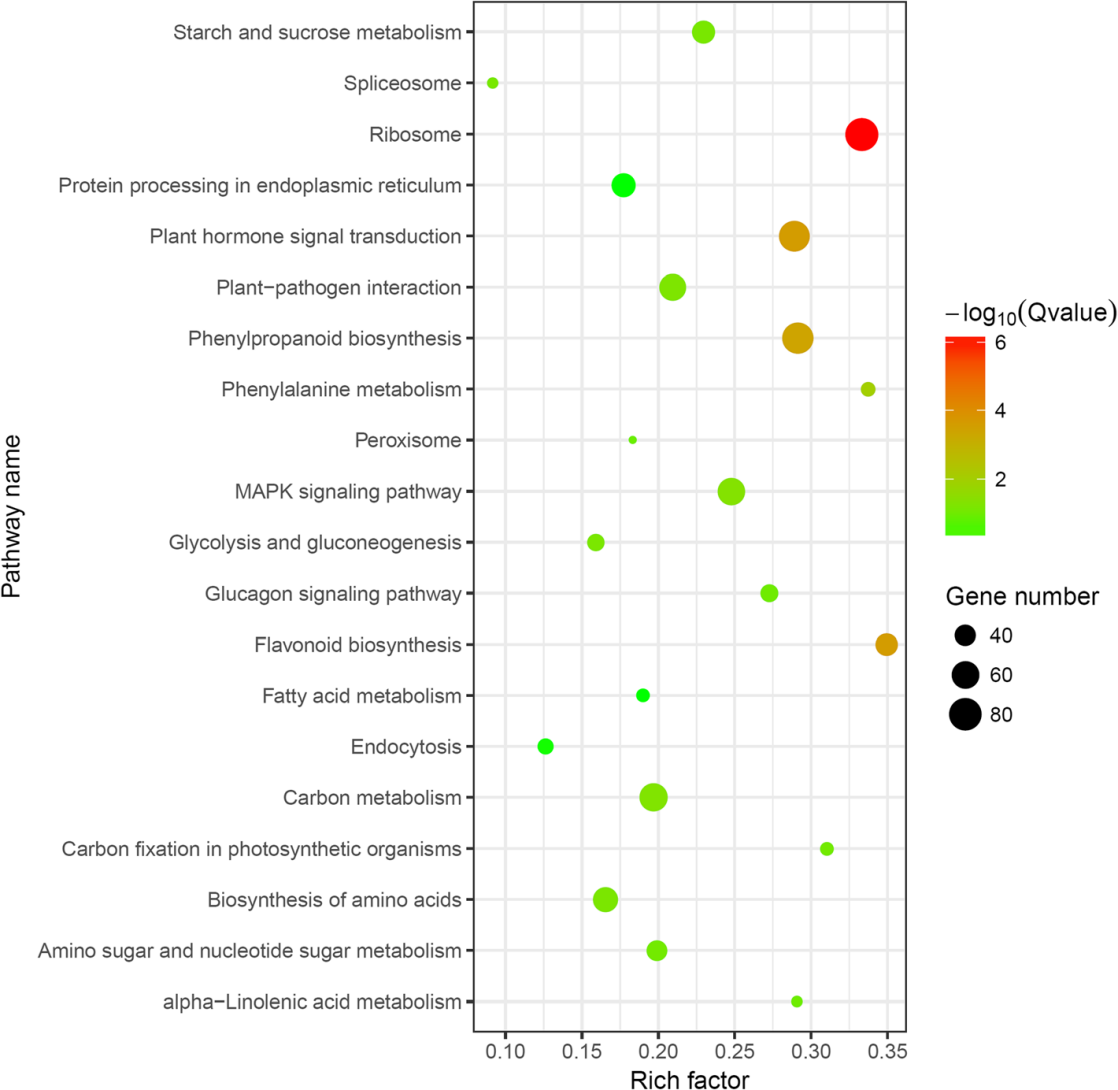
Top 20 pathways of KEGG functional enrichment among DEGs. Coloring indicates -log_10_(*Q* value) with higher in red and lower in green. And the lower *Q* value indicates the more significantly enriched. Point size indicates DEG number

Furthermore, 5599 of the significantly DEGs were blasted to the KEGG database to analyze their biological pathways. Among 205 of the enriched KEGG pathways (Additional file 3: Table S3), the most significant ones (*Q* ≤ 0.05) consist of “Ribosome”, “Phenylpropanoid biosynthesis”, “Plant hormone signal transduction”, “Flavonoid biosynthesis”, and “Phenylalanine metabolism” (Table 2 and Fig. 5), followed by others, such as “Carbon metabolism” (ko01200), “MAPK signaling pathway” (ko04016), “Plant-pathogen interaction” (ko04626), “Glucagon signaling pathway” (ko04922), “Biosynthesis of amino acids” (ko01230), and “Starch and sucrose metabolism” (ko00500).

**Table 2:** The significantly enriched pathways for DEGs in *G. biloba* ovules

Pathway	Pathway ID	Number of DEGs	*P* value	*Q* value
Ribosome	ko03010	82 (1.09%)	2.74E-09	7.43E-07
Flavonoid biosynthesis	ko00941	43 (0.57%)	2.69E-06	2.44E-04
Plant hormone signal transduction	ko04075	72 (0.95%)	2.70E-06	2.44E-04
Phenylpropanoid biosynthesis	ko00940	74 (0.98%)	6.07E-06	4.11E-04
Phenylalanine metabolism	ko00360	28 (0.37%)	2.13E-04	1.15E-02

### DEGs involved in hormone signal transduction pathway

**Fig. 6:**
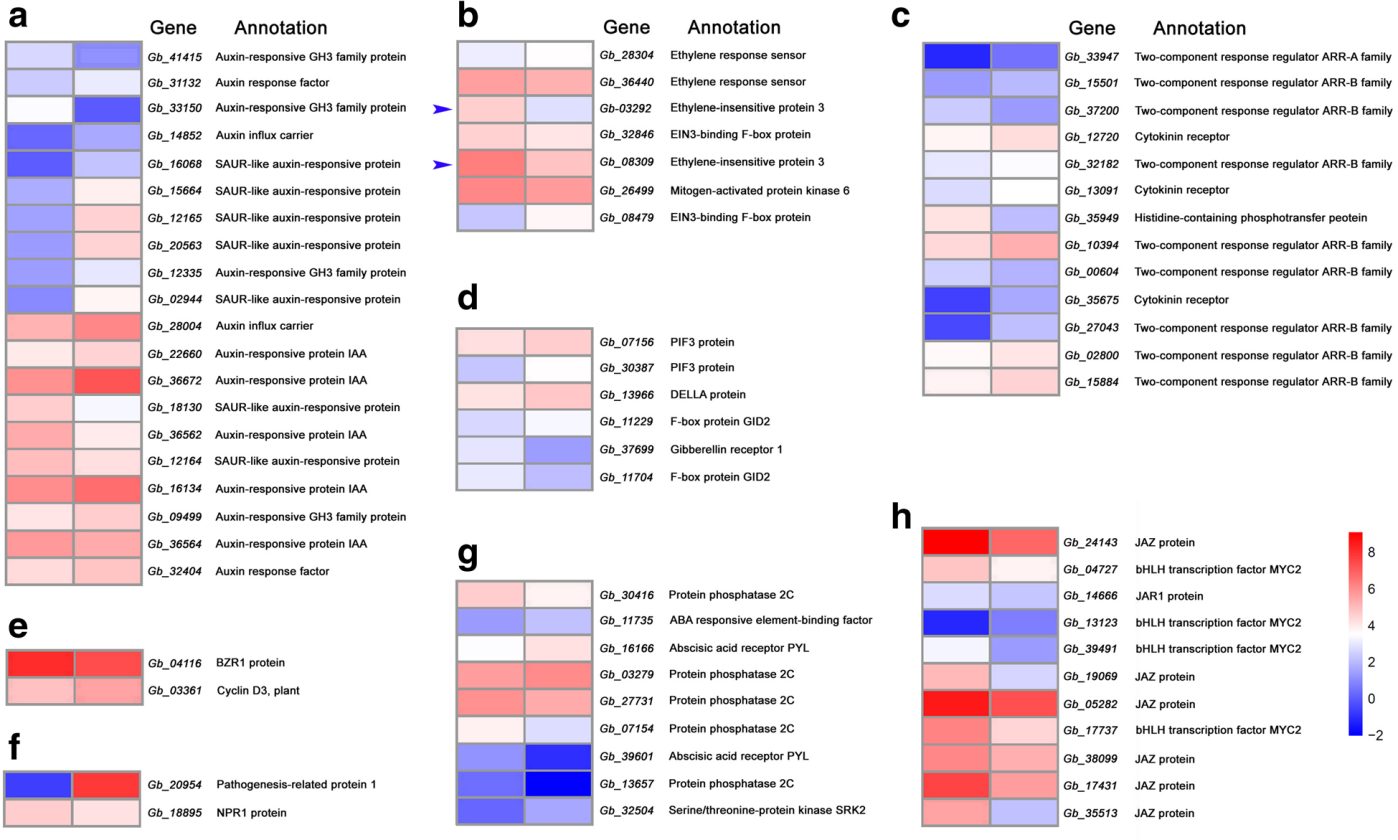
Heatmaps of expression patterns for DEGs involved in phytohormone signaling pathways, including auxin (**a**), ethylene (**b**), cytokinin (**c**), GA (**d**), BR (**e**), SA (**f**), ABA (**g**), and JA (**h**). Left and right box-columns represent pre-PCF and post-PCF libraries, respectively. Arrowheads indicate the significantly differentially expressed *EIN3* genes

Within the pathway of “Plant hormone signal transduction” (ko04075), there enriched 72 of the significantly DEGs (Table 3 and Additional file 4: Table S4), most of which were involved in auxin, cytokinin, ethylene, JA, ABA, GA, SA, and BR signaling pathways (Fig. 6). In the ethylene pathway, *ETR (Ethylene Receptor)*, *MPK6 (Mitogen-Activated Protein Kinase 6)*, *EIN3 (Ethylene-Insensitive Protein 3)*, and *EBF1/2 (EIN3-Binding F-Box Protein)*, have been documented to participate in the regulation of fruit ripening, senescence, and developmental PCD. In the present study, two orthologs of *EIN3* genes and one of *MPK6* gene were up-regulated in pre-PCF, compared to those in post-PCF, while both of two *ETR* or *EBF1/2* genes were inconsistent with each other in the transcript levels, respectively. In the auxin signaling pathway, both orthologs of two *AUX1* encoding for auxin influx carrier, and *ARF* for auxin response factor, were up-regulated in post-PCF, compared to those in pre-PCF. Moreover, there were 6 up- and 3 down-regulated transcripts belonging to *SAUR* genes, and 3 up-and 2 down-regulated ones encoding for auxin-responsive proteins (IAA), respectively. In the cytokinin pathway, 3 *CRE1* (encoding for cytokinin receptor), and 7 *ARR* (two-component response regulator), showed higher expression levels in post-PCF than in pre-PCF, while 1 *AHP* (histidine-containing phosphotransfer protein) was expressed in a reserve trend. In the pathway of JA signaling, a consistent down-regulation of 6 *JAZ* (encoding for jasmonate ZIM domain-containing protein), 4 *MYC2* (bHLH transcription factors), and 1 *JAR1* (jasmonic acid-amino synthetase), was presented. In the ABA pathway, *ABF* (encoding for ABA responsive element binding factor) and *SnRK2* (serine/threonine protein kinase), were up-regulated in post-PCF, compared to those in pre-PCF, while 4 *PP2C* (protein phosphatase 2C) were down-regulated. In the GA pathway, *GID1* (encoding for gibberellin receptor 1) was down-regulated, while both of *DELLA* and *PIF3* (phytochrome-interacting factor 3) were up-regulated in post-PCF, in contrast to those in pre-PCF, respectively. In addition, some DEGs involved in SA and BR signaling pathways were expressed in up- (*PR-1*, *CYCD3*) or down-regulated patterns (*NPR1*, *BZR1/2*), respectively.

**Table 3:** The DEGs involved in plant hormone signal transduction pathway in *G. biloba* ovules

Gene ID	KEGG Orthology	Signaling Pathway	Annotation
Gb_28304	K14509	Ethylene	ETR, ethylene response sensor
Gb_36440	K14509	Ethylene	ETR, ethylene response sensor
Gb_26499	K14512	Ethylene	MPK6, Mitogen-activated protein kinase 6
Gb_03292	K14514	Ethylene	EIN3, ethylene-insensitive protein 3
Gb_08309	K14514	Ethylene	EIN3, ethylene-insensitive protein 3
Gb_08479	K14515	Ethylene	EBF1/2, EIN3-binding F-box protein
Gb_32846	K14515	Ethylene	EBF1/2, EIN3-binding F-box protein
Gb_14852	K13946	Auxin	AUX1, auxin influx carrier
Gb_28004	K13946	Auxin	AUX1, auxin influx carrier
Gb_16134	K14484	Auxin	Auxin-responsive protein IAA
Gb_22660	K14484	Auxin	Auxin-responsive protein IAA
Gb_36562	K14484	Auxin	Auxin-responsive protein IAA
Gb_36564	K14484	Auxin	Auxin-responsive protein IAA
Gb_36672	K14484	Auxin	Auxin-responsive protein IAA
Gb_31132	K14486	Auxin	ARF, auxin response factor
Gb_32404	K14486	Auxin	ARF, auxin response factor
Gb_09499	K14487	Auxin	Auxin-responsive GH3 family protein
Gb_12335	K14487	Auxin	Auxin-responsive GH3 family protein
Gb_33150	K14487	Auxin	Auxin-responsive GH3 family protein
Gb_41415	K14487	Auxin	Auxin-responsive GH3 family protein
Gb_02944	K14488	Auxin	SAUR-like auxin-responsive protein
Gb_12164	K14488	Auxin	SAUR-like auxin-responsive protein
Gb_12165	K14488	Auxin	SAUR-like auxin-responsive protein
Gb_15664	K14488	Auxin	SAUR-like auxin-responsive protein
Gb_16068	K14488	Auxin	SAUR-like auxin-responsive protein
Gb_18130	K14488	Auxin	SAUR-like auxin-responsive protein
Gb_20563	K14488	Auxin	SAUR-like auxin-responsive protein
Gb_39828	K14488	Auxin	SAUR-like auxin-responsive protein
Gb_41374	K14488	Auxin	SAUR-like auxin-responsive protein
Gb_12720	K14489	Cytokinin	CRE1, cytokinin receptor
Gb_13091	K14489	Cytokinin	CRE1, cytokinin receptor
Gb_35675	K14489	Cytokinin	CRE1, cytokinin receptor
Gb_35949	K14490	Cytokinin	AHP, histidine-containing phosphotransfer protein
Gb_00604	K14491	Cytokinin	Two-component response regulator ARR-B family
Gb_02800	K14491	Cytokinin	Two-component response regulator ARR-B family
Gb_10394	K14491	Cytokinin	Two-component response regulator ARR-B family
Gb_15501	K14491	Cytokinin	Two-component response regulator ARR-B family
Gb_15884	K14491	Cytokinin	Two-component response regulator ARR-B family
Gb_27043	K14491	Cytokinin	Two-component response regulator ARR-B family
Gb_32182	K14491	Cytokinin	Two-component response regulator ARR-B family
Gb_37200	K14491	Cytokinin	Two-component response regulator ARR-B family
Gb_33947	K14492	Cytokinin	Two-component response regulator ARR-A family
Gb_04727	K13422	Jasmonic acid	bHLH transcription factor MYC2
Gb_13123	K13422	Jasmonic acid	bHLH transcription factor MYC2
Gb_17737	K13422	Jasmonic acid	bHLH transcription factor MYC2
Gb_39491	K13422	Jasmonic acid	bHLH transcription factor MYC2
Gb_14666	K14506	Jasmonic acid	JAR1, jasmonic acid-amino synthetase
Gb_05282	K13464	Jasmonic acid	JAZ, jasmonate ZIM domain-containing protein
Gb_17431	K13464	Jasmonic acid	JAZ, jasmonate ZIM domain-containing protein
Gb_19069	K13464	Jasmonic acid	JAZ, jasmonate ZIM domain-containing protein
Gb_24143	K13464	Jasmonic acid	JAZ, jasmonate ZIM domain-containing protein
Gb_35513	K13464	Jasmonic acid	JAZ, jasmonate ZIM domain-containing protein
Gb_38099	K13464	Jasmonic acid	JAZ, jasmonate ZIM domain-containing protein
Gb_11735	K14432	Abscisic acid	ABF, ABA responsive element-binding factor
Gb_16166	K14496	Abscisic acid	Abscisic acid receptor PYL
Gb_39601	K14496	Abscisic acid	Abscisic acid receptor PYL
Gb_03279	K14497	Abscisic acid	PP2C, protein phosphatase 2C
Gb_07154	K14497	Abscisic acid	PP2C, protein phosphatase 2C
Gb_13657	K14497	Abscisic acid	PP2C, protein phosphatase 2C
Gb_27731	K14497	Abscisic acid	PP2C, protein phosphatase 2C
Gb_30416	K14497	Abscisic acid	PP2C, protein phosphatase 2C
Gb_32504	K14498	Abscisic acid	SnRK2, serine/threonine-protein kinase SRK2
Gb_07156	K12126	Gibberellin	PIF3, phytochrome-interacting factor 3
Gb_30387	K12126	Gibberellin	PIF3, phytochrome-interacting factor 3
Gb_37699	K14493	Gibberellin	GID1, gibberellin receptor 1
Gb_13966	K14494	Gibberellin	DELLA protein
Gb_11229	K14495	Gibberellin	F-box protein GID2
Gb_11704	K14495	Gibberellin	F-box protein GID2
Gb_20954	K13449	Salicylic acid	PR1, pathogenesis-related protein 1
Gb_18895	K14508	Salicylic acid	regulatory protein NPR1
Gb_04116	K14503	Brassinosteroid	BZR1/2, brassinosteroid resistant 1/2
Gb_03361	K14505	Brassinosteroid	CYCD3, cyclin D3, plant

**DEGs Involved in the Regulation of PCD in** ***G. biloba*** **Ovules.**

To identify DEGs associated with the nucellar PCD during pollen chamber formation in *G. biloba* ovules, the functional annotations of DEGs were blasted against the Nr database. According to the resultant annotations, a total of 99 significantly DEGs related to PCD were identified and involved in different PCD processes including hormone signaling pathways, initiation, and execution (Additional file 5: Table S5). In the present study, ethylene signaling pathway was one of the significantly enriched KEGG pathways. Some genes were identified from the ethylene signaling pathway, including *MPK6*, *EIN3*, ethylene receptor gene *ETR*, and *EBF1/2*. Moreover, three DEGs involved in the process of ethylene biosynthesis were found out with significantly higher expression levels in the pre-PCF ovules than those in the post-PCF ones. Compared with their transcriptional amounts in the pre-PCF ovules, *Gb_41401* (encoding for 1-aminocyclopropane-1-carboxylate synthase, ACS), *Gb_08184* and *Gb_31357* (encoding for 1-aminocyclopropane-1-carboxylate oxidase, ACO) were down-regulated by 0.95-, 0.79-, and 3.30-fold in the post-PCF ovules, respectively (Additional file 5: Table S5 and Additional file 6: Figure S1). Furthermore, ethylene contents in between the two developmental ovules were manifested by a concomitant elevation in the pre-PCF ovules and a significant decrease in the post-PCF ones (Additional file 5: Table S5 and Additional file 6: Figure S1).

Downstream of ethylene signaling pathway, a distinct PCD-initiating and PCD-executing network is being coordinated to control the PCD process in a precise temporal and spatial pattern. In the study, some genes involved in the PCD initiation are mainly focused on the specific transcription factors (TFs), including *MYB*, *MADS-box*es, *bHLH*s, and *NAC*s. Other functional components, such as *CYTOCHROME C* (*CYTC-1*), *CALMODULIN* (*CAM*), *MAPK*, *LESION SIMULATING DISEASE 1* (*LSD1*), and calcium uniporter protein *MCU*, were also identified. A total of 45 DEGs was found out to be associated to calcium signaling (Additional file 7: Table S6), consisting of 15 calcineurin B-like proteins (*CBL*s), 4 calcium channels, 15 calmodulins (*CAM*s), and 11 calcium-dependent protein kinases (*CDPK*s). Moreover, a variety of genes associated with the PCD execution were enriched with two categories: protease and autophagy. The identified protease genes consist of *CYSTEINE PROTEINASE RD21A*, metacaspase (MC) genes *MC2* and *MC6*, *ASPARTIC PROTEASE*, *SENESCENCE-ASSOCIATED PROTEIN*, *VACUOLAR PROCESSING ENZYME* (*VPE*), serine-type Clp protease *CLPP*, *ENDOGLUCANASE*s *17* and *23-like*, *PECTINESTERASE*s *8* and *QUARTET 1* (*QRT1*), *XYLOGLUCAN ENDOTRANSGLUCOSYLASE/HYDROLASE*s *7* and *9*, exosome complex component *RRP45*, and Werner Syndrome-like exonuclease *WRNEXO*. On the other hand, autophagy-related genes comprise *AUTOPHAGY-RELATED PROTEIN*s *5* (*ATG5*), *8C* and *16–1*, syntaxin-related protein *KNOLLE*, protein-transport protein *SEC61B*, vesicle-transport protein *SEC22*, calnexin *CNX1*, calreticulin family protein *CALR* and *CALR3*. Additionally, some genes within the longevity regulating pathway were found out, including heat shock 70 kDa protein 1/2/6/8 (*HSC70–1*), Cu-Zn superoxide dismutase (*CSD1*), Fe-Mn superoxide dismutase (*FSD1 and MSD1*), catalase *CAT*, molecular chaperone *DnaK*, and chaperonin *GroEL*.

**Fig. 7:**
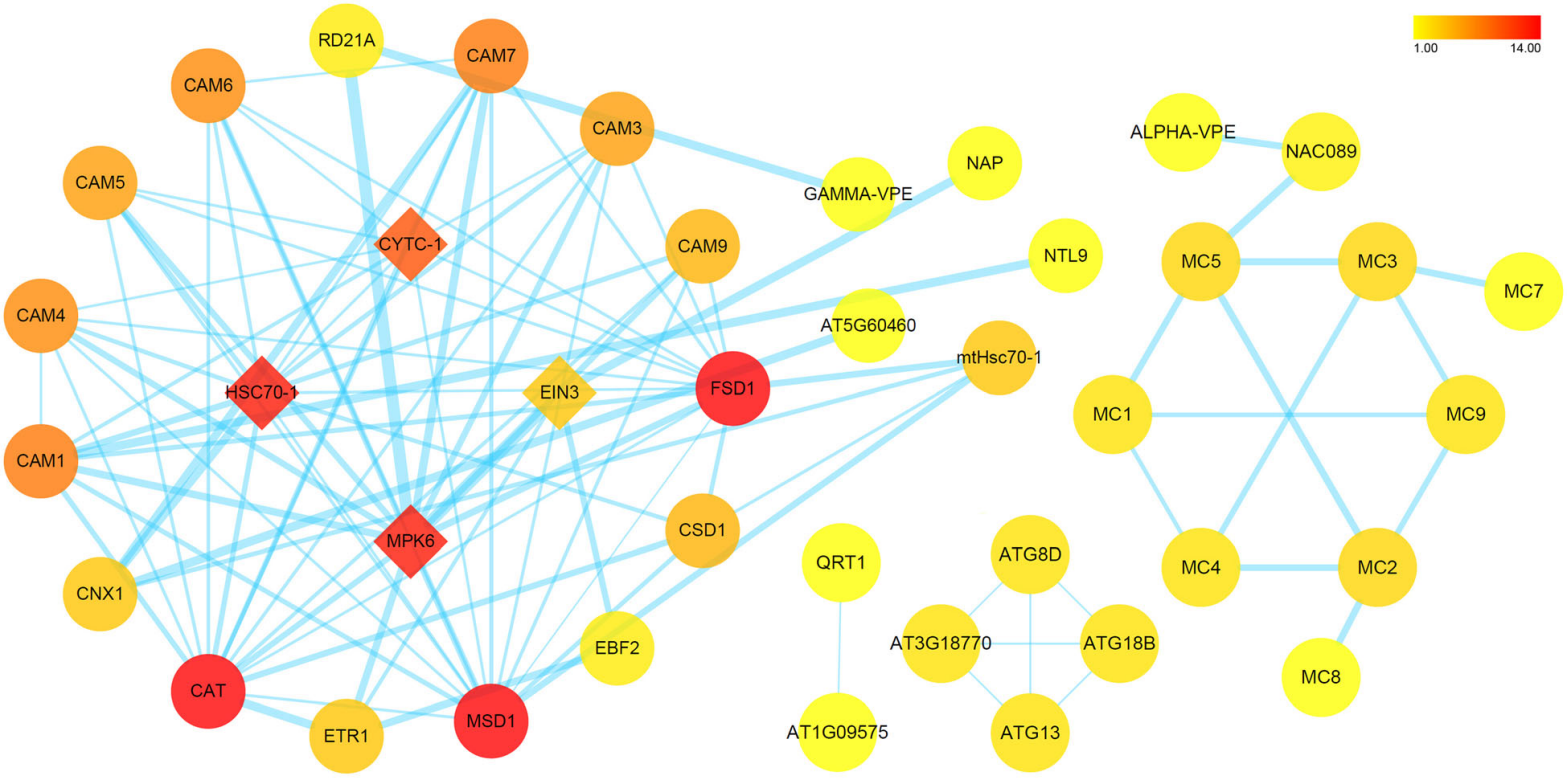
The protein-protein interaction network of the significantly DEGs involved in the nucellar PCD. The nodes represent target proteins shown by their gene names, respectively. The color band is illustrated from red to yellow in descending order of degree values. Edge size is mapped according to its corresponding parameter EdgeBetweenness with lower value to smaller size

To further investigate regulatory pathways of the nucellar PCD, a protein-protein interaction network was constructed for some significantly DEGs, based on their homologs from *A. thaliana* (Fig. 7). Within the resultant network, there presented two discrete pathways, albeit overlapping at some nodes. One is the MPK6-EIN3 network, containing some members involved in “Plant hormone signal transduction” (ko04075) pathway (Additional file 8: Figure S2). Noticeable, MPK6 has a strong interaction with RD21A and GAMMA-VPE. Another type of VPE, ALPHA-VPE, forms a protein-protein interaction network with various types of metacaspases (Fig. 7). And EIN3 has a direct interaction with NAP, a transcription factor of the NAC family involved in senescence. The other is the CYTC-1/HSC70–1 network. Within this network, both CYTC-1 and HSC70–1 could interact with a number of CAMs, as well as CAT, FSD1 and MSD1 (Fig. 7). In addition, CNX1, a calcium-binding protein, has interaction with AT5G60460 protein (a transport protein SEC61B), and CAM1 has interaction with NTL9 (a NAC transcription factor). Moreover, four autophagy-related proteins (ATG8D, ATG13, ATG18B, and AT3G18770) show cross-over interactions with each other. And pectinesterase QRT1, has interaction with AT1G09575 protein, a mitochondrial inner membrane calcium uniporter (Fig. 7).

### Expression profile analysis by RT-qPCR

**Fig. 8:**
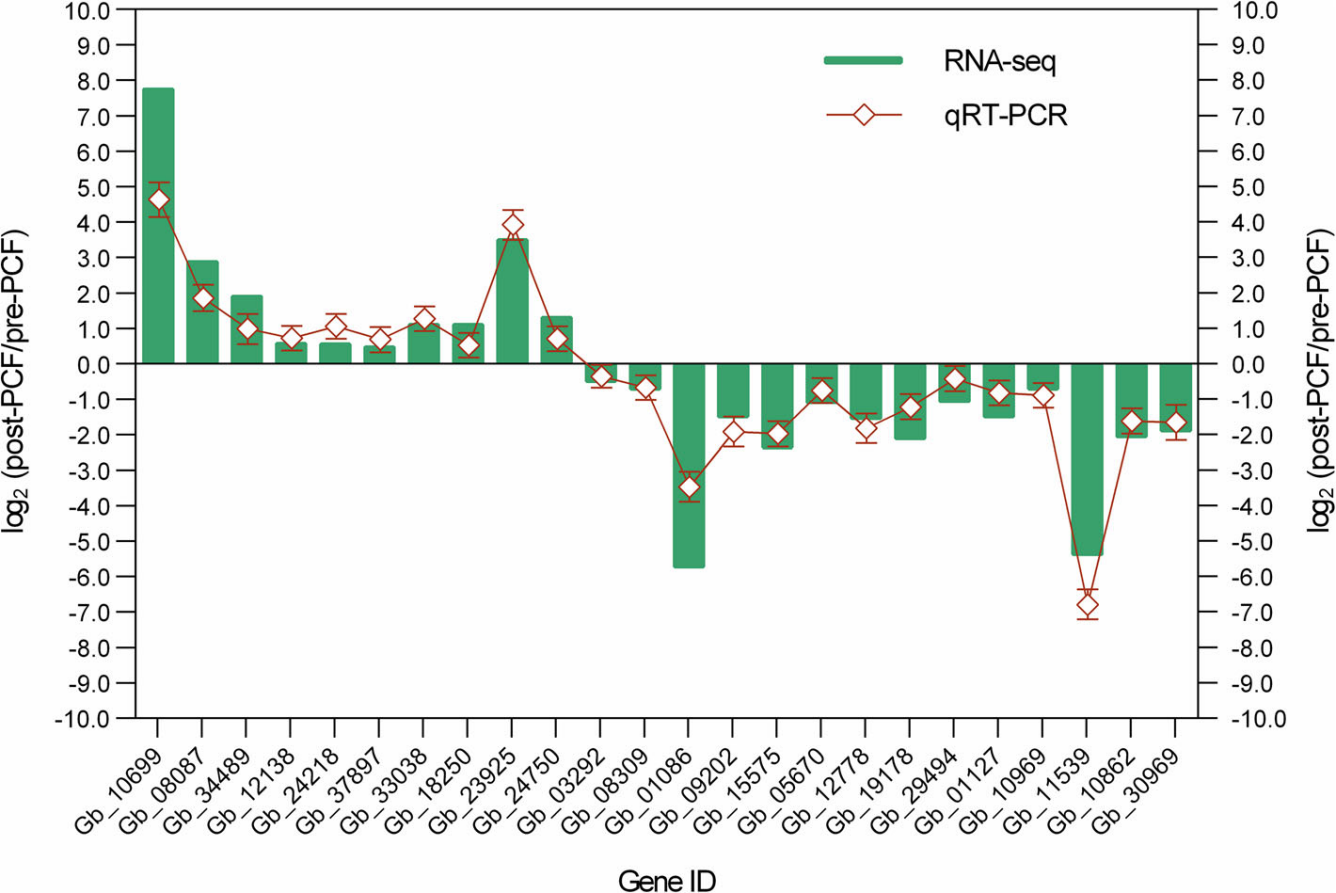
Expression level validation of DEG using qRT-PCR, in comparation to corresponding data detected in RNA-Seq. Relative expression ratio of each DEG is presented in a log_2_ value of post-PCF vs. pre-PCF libraries. The values are mean ± SE (*n* = 3)

10 of the up-regulated and 14 of the down-regulated DEGs were randomly selected for real-time quantitative PCR (RT-qPCR), to validate their expression levels during the process of nucellar PCD during pollen chamber formation in *G. biloba* ovules. Based on RT-qPCR results, it was found that the transcriptional levels of the tested genes were in a correlated trend with the respective abundance estimated by RNA-seq (Fig. 8), suggesting a relative rationality and accuracy of the transcriptome analysis in the present study.

### Subcellular localization of calcium within Nucellar cells

**Fig. 9:**
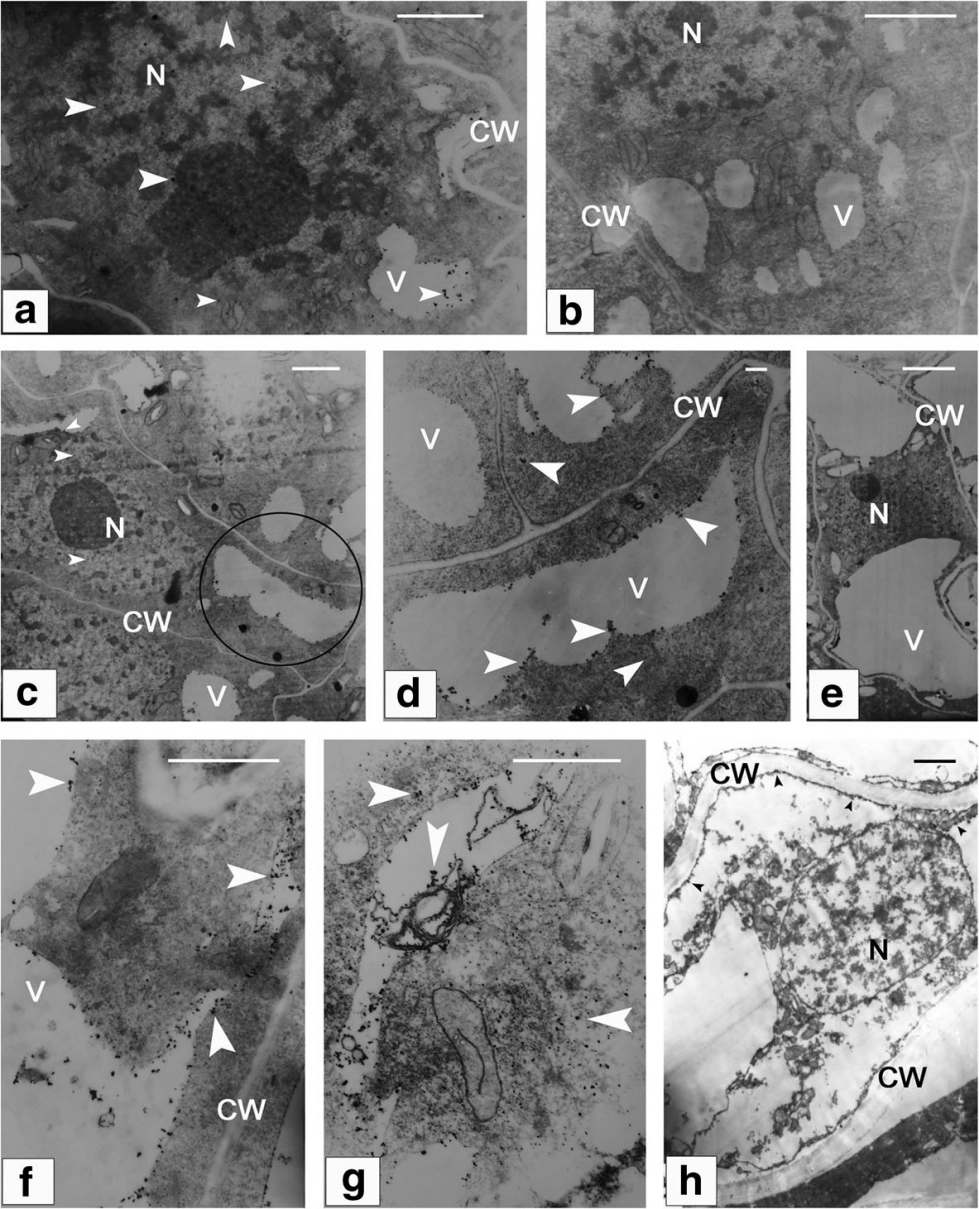
Subcellular localization of calcium within the nucellar cells involved in pollen chamber formation. (**a**) At the early stage of ovule development, a relatively higher Ca^2+^ precipitation is found in both of vacuoles and nucleus, compared with that in the cytoplasm. (**b**) A negative control of (a) without Ca^2+^ particles. (**c**), (**d**), (**f**), and (**g**) The nucellar cells at the micropylar end become elongated in shape, and distribution of Ca^2+^ precipitation is found to be increased in these nucellar cells. (**d**) Magnified view of the circled area in (**c**) shows numerous Ca^2+^ particles within the vacuole and cytoplasm. (**e**) A negative control of (**c**) without Ca^2+^ particles. (**g**) Deformed endoplasmic reticula are enclosed within the vacuole. (**h**) Few of Ca^2+^ particles are distributed along the cytoplasm debris, with no detectable aggregation of Ca^2+^ in the nucleus of the dying nucellar cell. Arrows indicate Ca^2+^ particles. Bars in (**a**), (**b**), (**c**), (**e**), and (**h**) = 2 μm, and in (**d**), (**f**), and (**g**) = 0.2 μm. CW, cell wall; N, nucleus; V, vacuole

Using in situ cytochemical method, dynamic changes in calcium concentration were visualized for the nucellar cells involved in pollen chamber formation (Fig. 9). At the early stage of ovule development, Ca^2+^ precipitation was aggregated in both of vacuoles and nucleus, in contrast to a scarce precipitation in the cytoplasm (Fig. 9a). Along with the developmental stage of ovule, an increased Ca^2+^ precipitation was found to be distributed in the elongated nucellar cell (Fig. 9c, d, f and g). In addition to their distribution in the vacuole (Fig. 9c and d), a large number of Ca^2+^ particles appeared in the cytoplasm (Fig. 9f and g). Some subcellular structures, such as endoplasmic reticula, were enclosed into the vacuole, showing a deformed shape and likely in a state of hydrolysis (Fig. 9g). For the negative control of corresponding nucellar cells, there were no visible Ca^2+^ particles (Fig. 9b and e). The amounts of detectable Ca^2+^ particles were significantly decreased in the dying nucellar cells. Within these nucellar cell, whose vacuole has been collapsed, few of Ca^2+^ particles were located along the cytoplasm debris and there presents no detectable aggregation of Ca^2+^ in the nucleus (Fig. 9h).

## Discussion

During the reproductive development, female *G. biloba* tree produces ovules, within which megaspores and female gametophyte are to be established for fertilization. Prior to the generation of female gametophyte, pollen grains have been pollinated onto the *G. biloba* ovules and stored in a cavity, namely pollen chamber, for around four months until completion of fertilization. Formation of pollen chamber is a natural process through the cell death of the specific nucellus at the early development of *G. biloba* ovules. Nucellar degeneration has been a developmental event widely existed in both gymnosperms and angiosperms [9]. Accumulated evidences have suggested that nucellar degeneration occurs by means of a developmental PCD [33, 36–38]. In the previous research, PCF concomitantly with the death of specific nucellar cells has been described as a timely and spatially regulated PCD process in *G. biloba* ovules [2–4]. However, little has been known about the molecular network controlling the nucellar PCD. Intensive genetic and genomic studies during the past decade have led to major advances in our understanding of animal and plant PCD at the molecular level. Base on the recent advances in regulatory mechanisms underlying diverse types of PCD, a comprehensive identification of DEGs and modulating pathways related to the nucellar PCD during PCF development was profiled using transcriptomic data via comparation between *G. biloba* ovules at the stages of pre- and post-PCF.

### Role of ethylene signaling pathway in nucellar PCD during PCF in *G. biloba* ovules

Phytohormone signaling has been thought as the common mechanism underlying upstream regulation of various dPCD for occurrence in a precise spatial and temporal way [8], including ethylene in aerenchyma formation and leaf senescence [17–19]; GA in tapetum PCD; auxin, cytokinin and BR in TE PCD [21, 22], respectively. In this study, KEGG-based analysis on the pathway enrichment of the DEGs showed that the pathway “Plant hormone signal transduction” (ko04075) was significantly enriched, in addition to those concerning the basic and secondary metabolisms. Noticeably, transcriptional expression of some key components within the ethylene signaling pathway, including *MPK6* and *EIN3*, were found to be significantly differential with their expression down-regulated in the post-PCF ovules compared to those in the pre-PCF ones. EIN3 has been shown to be involved in the trifurcate feed-forward pathway of age-dependent senescence and cell death [18]. According to the model for the EIN2–EIN3–NAC TFs regulatory cascade in the control of leaf senescence-associated PCD, EIN3 directly induces the expression of two key positive regulators of leaf senescence, i.e., NAC transcription factors ORE1 and AtNAP. Simultaneously, EIN3 directly suppresses the expression of miR164, which negatively regulates ORE1 at the post-transcriptional level [18, 19]. By analogy, the upregulated expression of *EIN3* in the pre-PCF ovules were likely to promote the nucellar PCD in this study. These results indicated that ethylene signaling pathway was activated for transcriptional regulation of the downstream targets at the stage of nucellar PCD preparation in *G. biloba* ovules. To investigate the ethylene signal involved in the PCF process, ethylene contents from the pre- and post-PCF ovules were assayed, respectively (Additional file 6: Fig. S1). It was found out that ethylene contents from the pre-PCF ovules were significantly higher than those in the post-PCF ones. And genes encoding for ACS and ACO, which play key roles in promoting ethylene biosynthesis, were significantly differentially expressed with a trend in accordance with that in ethylene contents (Additional file 5: Table S5 and Additional file 6: Figure S1), suggesting the molecular cues for the activated ethylene signaling pathway. With regard to the roles of other phytohormone, it is proposed that they should function as integrated regulators for developmental processes of whole ovules, such as cell division, elongation, and enlargement, etc., based on the pathway “Plant hormone signal transduction” in KEGG database. A set of differentially expressed genes within various types of hormone signaling, has been demonstrated during the nucellar PCD in *G. biloba* ovules (Table 3 and Additional file 4: Table S4), indicating that there might existed an interaction effect of ethylene and other hormones on the nucellar PCD. During the process of aleurone cell death, it has been reported that GA and ABA are in an antagonistic way to regulate its degeneration [8]. The antagonistic effects have also been suggested during leaf senescence and xylogenesis, respectively [20, 22]. For instance, SA, ABA, JA and ethylene promote leaf senescence, while cytokinin, GA, and auxin delay its senescence [20]. As showed in Fig. 6, gens within ethylene signaling, such as *ETR* (*Gb_36440*), *EIN3* (*Gb_03292* and *Gb_08309*), and *MPK6* (*Gb_26499*), were downregulated in their expression levels compared between post-PCF and pre-PCF. On the contrary, some genes involved in auxin pathway, such as *AUX1* (*Gb_14852* and *Gb_28004*), SAUR-like auxin-responsive protein (*Gb_02944*, *Gb_12164*, *Gb_12165*, *Gb_15664*, *Gb_16068*, and *Gb_20563*), Auxin-responsive protein (*Gb_16134*, *Gb_22660*, *Gb_36672*, and *Gb_32404*) or in cytokinin pathway, like *CRE1* (*Gb_12720* and *Gb_13091*), and two-component response regulator ARR-B family (*Gb_15884*, *Gb_02800*, and *Gb_10394*) were upregulated. Therefore, it is feasible that nucellar PCD process in *G. biloba* ovules should mainly be controlled by ethylene, antagonistically interacting with other acting hormones (auxin and cytokinin).

### Initiation and execution in nucellar PCD during PCF in *G. biloba* ovules

Various key TFs have been described as bridges linking hormone signaling with PCD control [7, 8]. In the present study, several DEGs encoding for homologs of the specific TFs associated with particular PCD have been identified, including one *MYB*, two *MADS-box*es, six *bHLH*s, and thirteen *NAC*s. All of these TF genes have exhibited a similarity in the expression trend with significantly higher levels in the pre-PCF than those in the post-PCF. Homologs of MYB [39] and bHLH [35] have been known for involvement in promoting tapetum PCD, respectively. MADS-box was found to promote nucellar PCD during rice seed development [40]. And some NAC TFs function downstream of ethylene signaling pathway to modulate the cascade of leaf senescence-associated PCD [41]. Altogether, these results suggested that downstream of hormone signaling, several TFs should play important roles in ensuring nucellar PCD initiation and execution during PCF in *G. biloba* ovules.

Changes in cytoplasmic levels of calcium and cytochrome *c* have been proposed as parts of dPCD signaling network [42, 43]. During apoptosis in animals, once released from the mitochondria, cytochrome *c* associates with cytoplasmic Apaf1 to form the apoptosome, a large complex that processes initiator caspases [44]. Several plant PCD cases, such as the floral organ senescence in petunia and the embryonic suspensor cell death in runner bean (*Phaseolus coccineus*), are accompanied by release of mitochondrial cytochrome *c* [37, 42]. In the present case of nucellar PCD, the transcript amounts of genes encoding for cytochrome *c* and calmodulin were significantly higher in the pre-PCF ovules compared to those in the post-PCF ones. And within the constructed protein-protein interaction network, CYTC-1 showed a multiple interaction with various CAMs (Fig. 7). The activities of a large number of enzymes and other proteins are under the control of CAM-Ca^2+^ complex. Ca^2+^ signaling has been documented to be associated with various PCD cases, including regulating activities of Ca^2+^-dependent endonucleases and hydrolytic enzymes [8, 45–47]. Cytoplasmic Ca^2+^-influx from vacuoles and endoplasmic reticulum may be an early event in the PCD regulation pathway [48]. In the present research, 45 DEGs associated to calcium signaling were identified, including *CBL*s for calcium sensors, *MCU* for calcium channel, *CAM*s and *CDPK*s for calcium-binding proteins. Although some of them were not significant DGEs, these genes were actively expressed and thus were functional within calcium signaling pathway (Additional file 5: Table S5 and Additional file 7: Table S6). With regard to calcium-binding protein CAM, its upregulated expression, along with the differential expression of other calcium sensors and *MCU* for calcium uniporter protein, suggests that cascade events are likely to contribute to the calcium signaling. Moreover, elevated calcium levels been observed in the nucellar cells undergoing PCD in *G. biloba* ovules (Fig. 9). Therefore, it is reasonable to infer that cytochrome *c* and calcium should be related to modulating the nucellar PCD initiation in *G. biloba* ovules. Despite both the dynamic changes in calcium influx and significantly DEGs associated to Ca^2+^ signaling, including CAM, CBL, and CDPK, were identified during the nucellar PCD in *G. biloba* ovules, downstream cascades within Ca^2+^ regulation pathway in the PCD process are still unknown. A Ca^2+^/Mg^2+^-dependent nuclease was reported to be involved in both wheat aleurone and nucellus cells undergoing PCD [47]. The significantly DEGs encoding for various proteinases have been showed in the present study. Whether their biological functions or activities in the nucellus PCD are proceed through the Ca^2+^-mediated regulatory network, provides a point for future research.

During the PCD execution in this study, the significantly DEGs encoding for a variety of proteases have been identified, including cysteine proteinases *RD21A*, *MC2* and *MC6*, *ASPARTIC PROTEASE*, *VPE*, *SENESCENCE-ASSOCIATED PROTEIN*, *ENDOGLUCANASE*s *17* and *23-like*, *PECTINESTERASE*s *8* and *QRT1*, *XYLOGLUCAN ENDOTRANSGLUCOSYLASE/HYDROLASE*s *7* and *9*, *RRP45* and *WRNEXO*. In addition to mediating ethylene signaling, MPK6 could strongly interact with RD21A and GAMMA-VPE (Fig. 7 and Additional file 8: Figure S2), both of which are members of cysteine proteinase, and thought to be associated with several types of cell death. And ALPHA-VPE has interaction with various types of MCs mediated by a NAC TF (Fig. 7). These results suggest that there exist regulatory networks among these components. The identified MCs, VPE, and other proteases, might function as their homologs known to have a principal role in proceeding to degrade many essential cellular targets [26, 31, 32, 34, 49]. Endoglucanases, pectinesterases, and xyloglucan endotransglucosylase/hydrolases, might be associated with recycling of carbohydrates in dying cells. And homologs of exosome complex component RRP45 and Werner Syndrome-like exonuclease WRNexo are likely to participate in DNA degradation [50].

Apart from the effects of proteases, autophagy occurs concomitantly with the execution of nucellar PCD in *G. biloba* ovules. Autophagy is a highly regulated process during which cytoplasmic materials are enclosed in double-membrane-bound vesicles that are then targeted to the vacuole or lysosome for degradation [51]. Unlike those of animals, vacuoles participate in the autolysis process of plant PCD [45, 52]. In the previous research on nucellar PCD in *G. biloba* ovules, ultrastructural observation clearly showed that some double-membrane bodies were being engulfed in the vacuole, as well as a convoluted membrane structure fusing with the vacuole [3]. In accordance with the observation, autophagy-related genes, including *AUTOPHAGY-RELATED PROTEIN*s *5*, *8C* and *16–1*, *KNOLLE*, *SEC61B*, *SEC22*, *CNX1*, *CALR* and *CALR3*, are found to be differentially expressed (Additional file 1: Table S1).

## Conclusions

**Fig. 10:**
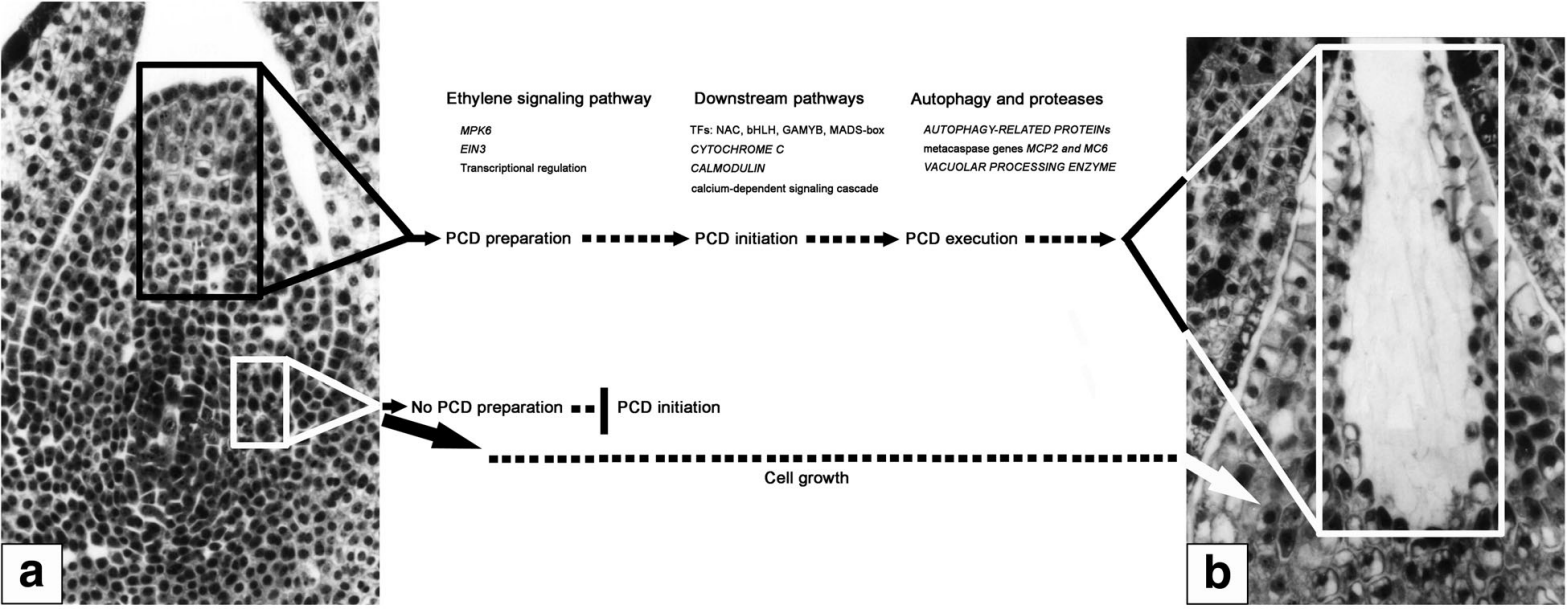
Tentative model for the nucellar PCD during pollen chamber formation in *G. biloba* ovules. Ovules at the stage of pre-PCF (**a**) and post-PCF (**b**) are sectioned longitudinally. The black-boxed area in (**a**) contains nucellar cells at the micropylar end of ovule, which are to undergo PCD to generated a pollen chamber, bounded by the white-box in (**b**). Other nucellar cells, represented by a white-boxed area in (**a**), keep growth throughout the stages of PCF

Transcriptomic profiling unravels the DEGs and modulating pathways with respective to the nucellar PCD during pollen chamber formation in *G. biloba* ovules. Based on these results, a putative working model, consisting of three overlapping processes, is proposed (Fig. 10): at the stage of PCD preparation, ethylene signaling pathway is activated for transcriptional regulation of the downstream targets; subsequently, at the stage of PCD initiation, the upregulated expression of several TFs, i.e., *NAC*, *bHLH*, *MADS-box*, and *MYB*, further promotes the corresponding transcript levels of *CYTOCHROME C* and *CAM*s, thereby, leads to the PCD initiation via the calcium-dependent signaling cascade; finally, at the stage of PCD execution, some proteases like MCs and VPE for hydrolysis, together with the process of autophagy, play roles in the clearance of cellular components. Afterwards, a pollen chamber is generated from the removal of specific nucellar cells in the developing ovule.

## Methods

### Plant materials

**Fig. 11:**
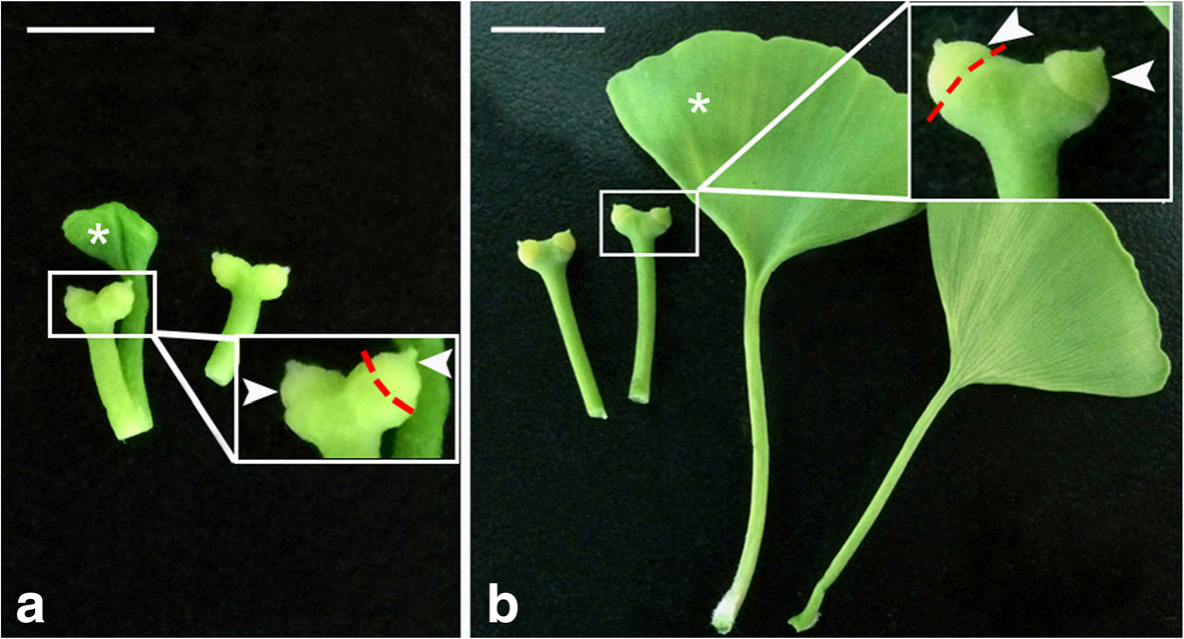
Ovule collection from *G. biloba* strobili at the stage of pre-PCF (**a**) or post-PCF (**b**). Magnified view of the boxed area shows ovules (arrowheads) collected from megasporophyll, and red-dash lines marking the junction region between ovules and megasporophyll. *G. biloba* leaves (asterisks) are positioned along with ovules. Bars = 1 cm

The ovules were collected from the same cultivar, *Ginkgo biloba* ‘Foshou’, at the campus of Anhui Agricultural University, Hefei, China. Sampling were performed every two days from 17th of March to 15th of April in 2016, respectively. The progressive development of pollen chamber in ovules were determined by micro-sections prepared in Epon 812. According to the ovule sections (Fig. 1 and Additional file 9: Figure S3), nucellar cells in the collected ovules on the 17th of March, were uniform in a rounded morphology and no occurrence of longitudinally elongation of nucellar cells (Fig. 1a and Additional file 9: Figure S3a and S3b), whereas nucellar cells at the micropylar end in those collected on the 1st of April, have been removed and a cavity of individual pollen chamber has been generated (Fig. 1c and Additional file 9: Figure S3e). Therefore, the ovules (collected on the 17th of March) at the stage of Fig. 1a (pre-PCF) and ones (on the 1st of April) at the stage of Fig. 1a (post-PCF), with three biological replicates, were used as samples for RNA-seq in this study (Fig. 11). In order to collect the right stage sample without microscope, the petiole length of megasporophyll where *G. biloba* ovules are situated were measured. Subsequently, a correlationship between the petiole length and the developmental stage of ovules was used as the criterion for sampling respective ovules. As showed in Additional file 9: Figure S3, ovules at pre-PCF, PCF in progress (Additional file 9: Fig. S3c and S3d), and post-PCF, had 0.60 ± 0.11 cm, 1.40 ± 0.16 cm, and 2.10 ± 0.17 cm on average in petiole length of megasporophyll, respectively. The climate data from March 10th to April 15th in 2016 were collected in Additional file 10: Table S7. As referred to the Additional file 10: Table S7, there is no occurrence of adverse weather condition during the period of sampling *G. biloba* ovules.

### DGE library construction and Illumina sequencing

After grinding into powder in liquid nitrogen, ovules from each sample were applied to total RNA extraction, followed by isolating mRNAs. The individual cDNA was synthesized using the random hexamers as primers and mRNA templates. The resultant products were connected with adapters, followed by size selection and PCR amplification. The constructed library was analyzed by Illumina HiSeq™ 4000 sequencing platform (BGI, Shenzhen, China). The data of RNA-Seq have been assigned with the accession number SRP158368 in NCBI (http://www.ncbi.nlm.nih.gov/sra).

### Analysis of differentially expressed gene and Functional annotation

Adaptor sequences and low-quality reads were initially filtered from the raw data. Then the remaining ones, called as clean reads, were aligned to the reference genome of *G. biloba* [53], using HISAT2 program [54]. Subsequently, unigene expression was calculated as the FPKM (fragments per kilo bases of exons for per million mapped reads) with the software package Cufflinks [55]. For both the pre-PCF and post-PCF libraries, the FPKM values were applied to measuring the log_2_ ratios (post-PCF/pre-PCF). And DEGs were filtered by a *FDR* (false discovery rate) value ≤0.05. Gene annotation were performed throughout blasting against the local Nr database (downloading from http://ftp-private.ncbi.nlm.nih.gov). Both annotations of GO (http://www.geneontology.org/) and KEGG (http://www.genome.jp/kegg/) were carried out to identify functional genes. Subsequently, functional enrichment of GO and KEGG pathway were analyzed using R package PHYPER, respectively. The terms were considered to be significantly enriched if *FDR* ≤ 0.001.

### Assay for ethylene content in *G. biloba* ovules

*G. biloba* ovules (0.25 g) were collected as mentioned above. Ethylene contents from the pre- and post-PCF ovules were assayed on a Varian 3800 gas chromatograph (Walnut Creek, CA, USA), using the previous method by Wang et al. [56]. The method validation followed the control procedures for the prepared ethylene concentration (10 μL/L).

### Construction and analysis of protein-protein interaction network

Protein-protein interactions were searched through the STRING (https://string-db.org/cgi). The proteins, putatively encoded by the significantly DEGs involved in the nucellar PCD, were mapped to the network according to information of their respective homologs from *Arabidopsis thaliana*. The protein interaction network was visualized using the software Cytoscape [57].

### qRT-PCR analysis

qRT-PCR was performed using CFX96 Touch™ System (BIO-RAD, USA), with the parameters: 96 °C for 2 min, followed by cycling for 30 rounds (96 °C for 10 s, 58 °C for 10 s, and 68 °C for 30 s). To calculate the expression abundances of target genes, the method 2^-ΔΔCt^ for statistics was applied with three biological replicates [58], and the *G. biloba* gene *GAPDH* was taken as an internal reference. All primer pairs for these qRT-PCR were deposited in Additional file 11: Table S8.

### Microscope observation and calcium-Cytochemical localization

The sections (8 μm in thickness) of the collected *G. biloba* ovules were prepared using paraffin-cutting and stained with safranine O for light microscope observation. Calcium-cytochemical localization in *G. biloba* nucellar cells was visualized by producing precipitation with potassium pyroantimonate, according to the previous method [4]. Observation and photographs were taken under JEM-100CX transmission electron microscope.

## Additional files

Additional file 1: **Table S1.** All the identified DEGs between pre-PCF and post-PCF libraries. (XLSX 1565 kb)

Additional file 2: **Table S2.** GO enrichment analysis of differentially expressed genes in *G. biloba* ovules. (XLSX 196 kb)

Additional file 3: **Table S3.** KEGG pathway analysis of differentially expressed genes in *G. biloba* ovules. (XLSX 33 kb)

Additional file 4: **Table S4.** The DEGs involved in plant hormone signal transduction pathway. (XLSX 20 kb)

Additional file 5: **Table S5.**The significantly DEGs involved in the nucellar PCD during pollen chamber formation in *G. biloba* ovules. (XLSX 39 kb)

Additional file 6: **Figure S1.** Transcriptional expression levels of the genes involved in ethylene biosynthesis (upper panel) and ethylene contents (lower panel) in pre- and post-PCF *G. biloba* ovules. Abundance of gene transcripts was presented by FPKM value resulted from the DEGs analysis in this study. Ethylene contents were normalized as nmol per gram fresh weight (Fw). (TIF 141 kb)

Additional file 7: **Table S6.** The DEGs associated to calcium signaling in *G. biloba* ovules. (DOCX 16 kb)

Additional file 8: **Figure S2.** The identified DEGs involved in plant hormone signal transduction by KEGG enrichment. (TIF 446 kb)

Additional file 9: **Figure S3.** A correlationship between the petiole length of megasporophyll and the developmental stage of ovules. Ovules at the developmental stages of pre-PCF (a and b), PCF in progress (c and d), and post-PCF (e) were determined by micro-sections from the representative samples and observed under microscope. The petiole length was a mean value of 10 megasporophylls randomly selected from one set of ovule samples, prior to the preparation for their ovule sections. Bars = 250 μm. Abbreviations: I, integument; M, micropyle; Nu, nucellus. (TIF 527 kb)

Additional file 10: **Table S7.** The climate data from March 10th to April 15th in 2016. (XLSX 28 kb)

Additional file 11: **Table S8.** The primers of DEGs for RT-qPCR in *G. biloba* ovules. (XLSX 24 kb)

## Abbreviations

ATGs: autophagy-related proteins
CAMs: calmodulins
DEGs: differentially expressed genes
EBF1/2: EIN3-binding F-box protein
EIN3: ethylene-insensitive protein 3
ETR: ethylene receptor
GO: Gene Ontology
KEGG: Kyoto Encyclopedia of Genes and Genomes
MCs: metacaspases
MPK6: mitogen-activated protein kinase 6
PCD: programmed cell death
PCF: pollen chamber formation
VPE: vacuolar processing enzyme
