# Supplementary figures and images for: Transcriptomic profiling identifies differentially expressed genes associated with programmed cell death of nucellar cells in Ginkgo biloba L
Source: BMC Plant Biol. 2019 Feb 28;19:91. doi: 10.1186/s12870-019-1671-8 (PMC6396491; doi:10.1186/s12870-019-1671-8)

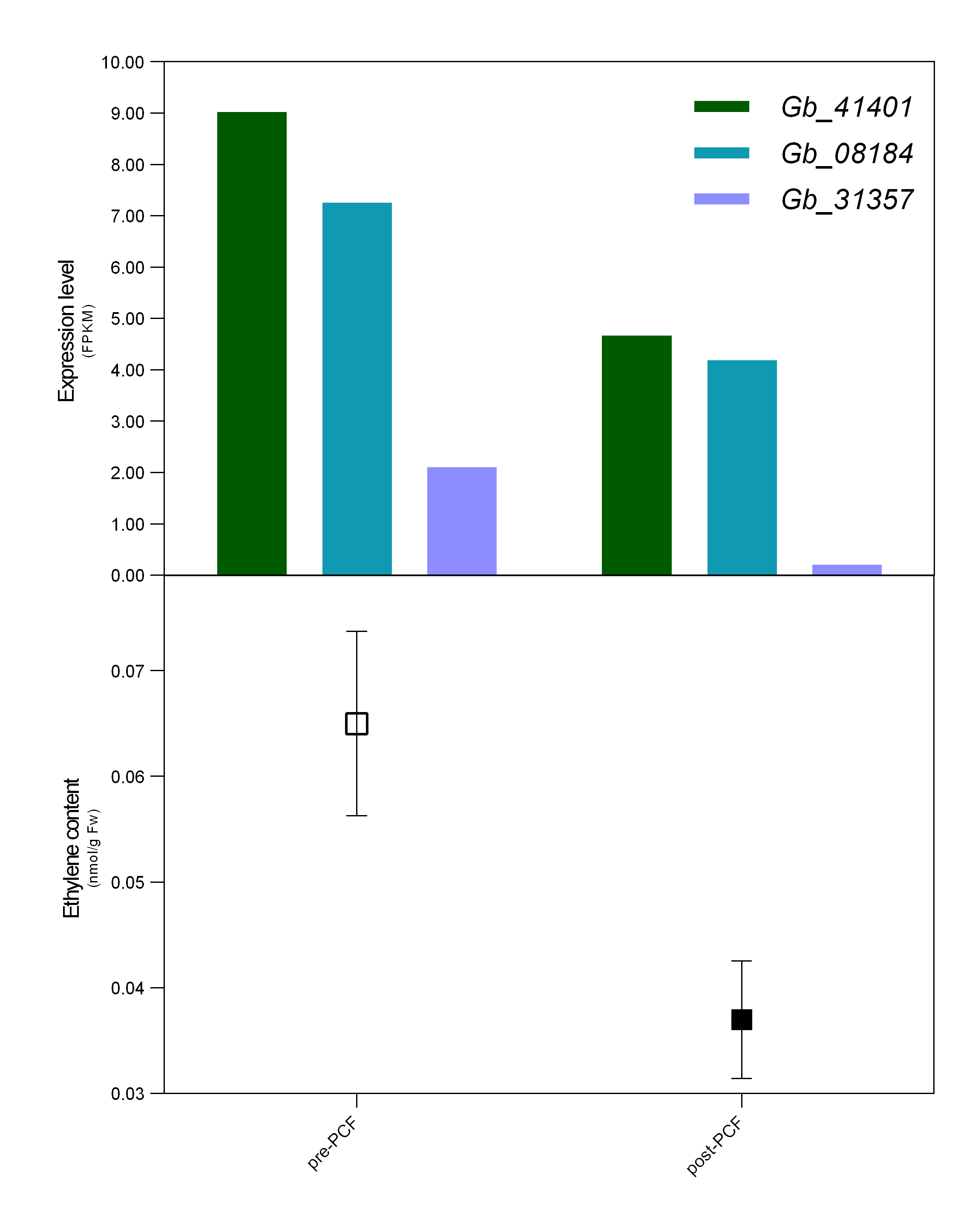

Supplement: Supplementary file 6 — Figure S1. Transcriptional expression levels of the genes involved in ethylene biosynthesis (upper panel) and ethylene contents (lower panel) in pre- and post-PCF G. biloba ovules. Abundance of gene transcripts was presented by FPKM value resulted from the DEGs analysis in this study. Ethylene contents were normalized as nmol per gram fresh weight (Fw). (TIF 141 kb) [file 12870_2019_1671_MOESM6_ESM.tif]

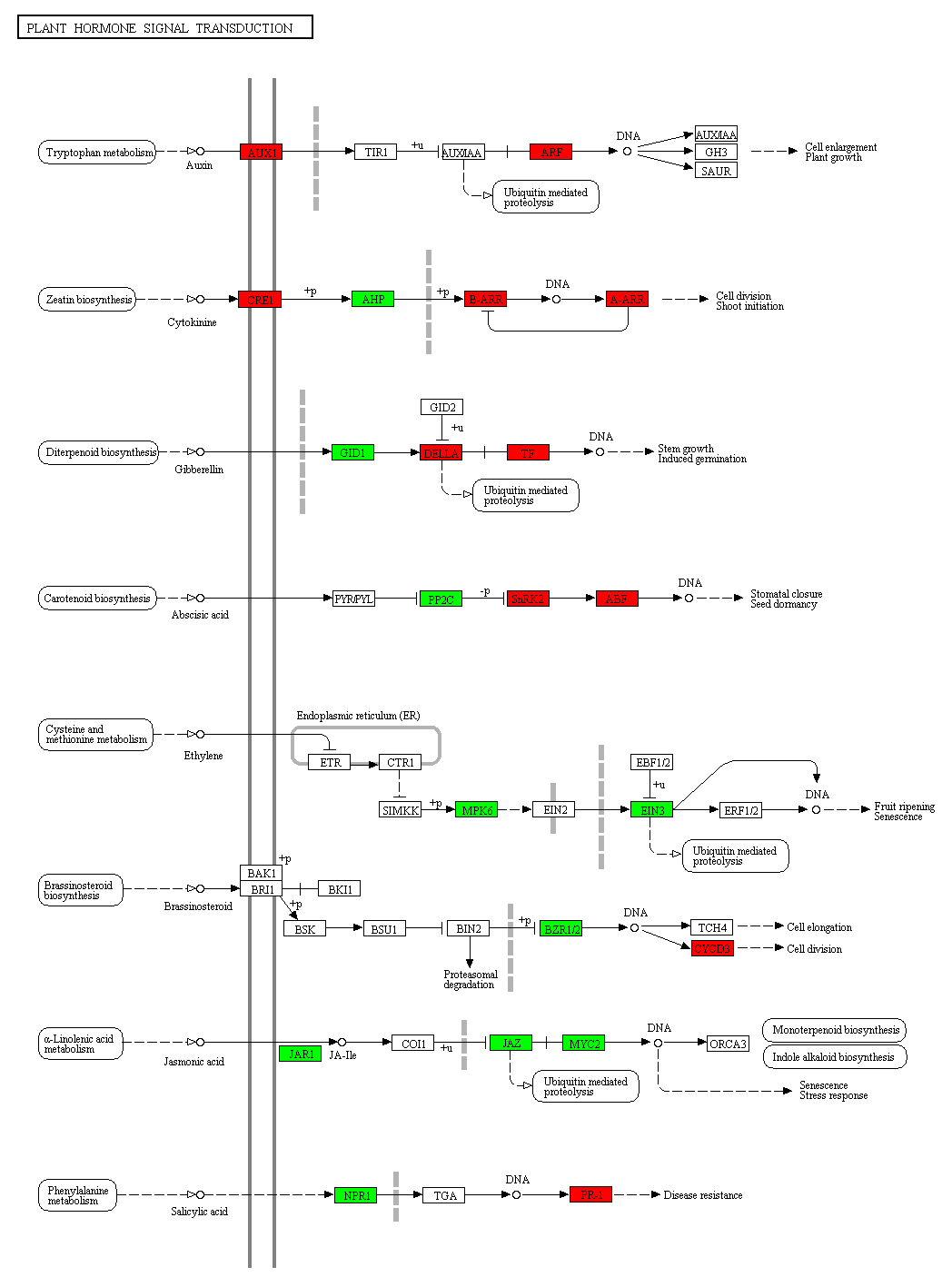

Supplement: Supplementary file 8 — Figure S2. The identified DEGs involved in plant hormone signal transduction by KEGG enrichment. (TIF 446 kb) [file 12870_2019_1671_MOESM8_ESM.tif]

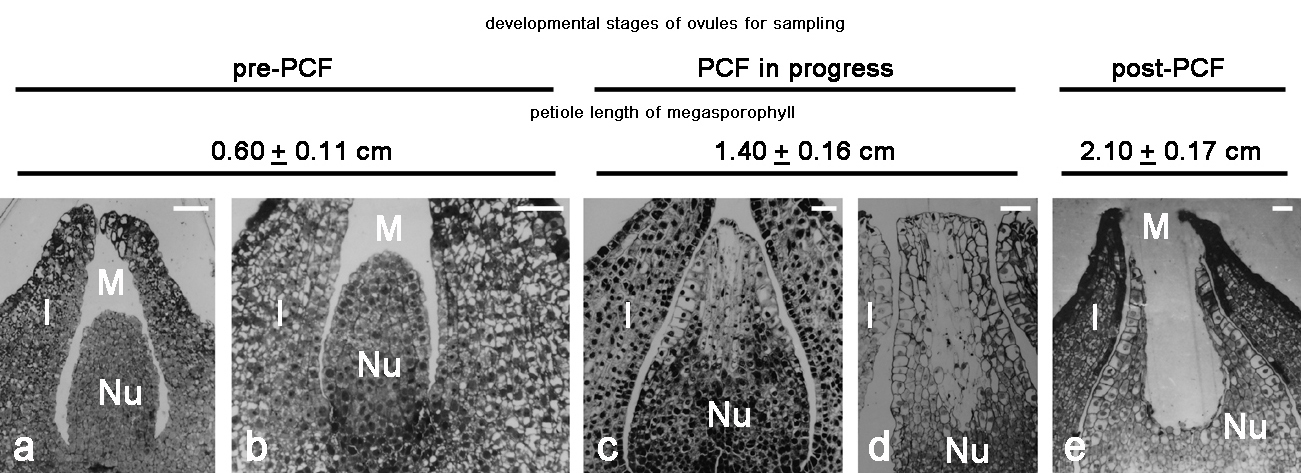

Supplement: Supplementary file 9 — Figure S3. A correlationship between the petiole length of megasporophyll and the developmental stage of ovules. Ovules at the developmental stages of pre-PCF (a and b), PCF in progress (c and d), and post-PCF (e) were determined by micro-sections from the representative samples and observed under microscope. The petiole length was a mean value of 10 megasporophylls randomly selected from one set of ovule samples, prior to the preparation for their ovule sections. Bars = 250 μm. Abbreviations: I, integument; M, micropyle; Nu, nucellus. (TIF 527 kb) [file 12870_2019_1671_MOESM9_ESM.tif]
