# Supplementary material for: Transcriptomic profiling identifies differentially expressed genes associated with programmed cell death of nucellar cells in Ginkgo biloba L
Source: BMC Plant Biol. 2019 Feb 28;19:91. doi: 10.1186/s12870-019-1671-8 (PMC6396491; doi:10.1186/s12870-019-1671-8)
Supplement: Supplementary file 7 — Table S6. The DEGs associated to calcium signaling in G. biloba ovules. (DOCX 16 kb) [file 12870_2019_1671_MOESM7_ESM.docx]

| **Table S6** The DEGs associated to calcium signaling in *G. biloba* ovules. | | | |
| --- | --- | --- | --- |
| **Gene ID** | **log2 Ratio (post-PCF/pre-PCF)** | **Significant** | **Annotation** |
| Gb_02215 | -0.0344235 | no | CBL, calcineurin B-like protein |
| Gb_02303 | -0.0933278 | no | CBL, calcineurin B-like protein |
| Gb_04320 | 0.629254 | yes | CBL, calcineurin B-like protein |
| Gb_09743 | 0.233603 | no | CBL, calcineurin B-like protein |
| Gb_09744 | 0.0445673 | no | CBL, calcineurin B-like protein |
| Gb_19203 | -0.1588 | no | CBL, calcineurin B-like protein |
| Gb_19459 | -2.4201 | yes | CBL, calcineurin B-like protein |
| Gb_19683 | 0.642567 | yes | CBL, calcineurin B-like protein |
| Gb_20846 | 0.426017 | no | CBL, calcineurin B-like protein |
| Gb_24001 | 1.41293 | yes | CBL, calcineurin B-like protein |
| Gb_24263 | 0.412654 | no | CBL, calcineurin B-like protein |
| Gb_27095 | -0.27619 | no | CBL, calcineurin B-like protein |
| Gb_31158 | -2.48311 | yes | CBL, calcineurin B-like protein |
| Gb_33410 | -0.0506206 | no | CBL, calcineurin B-like protein |
| Gb_41456 | 0.223248 | no | CBL, calcineurin B-like protein |
| Gb_35768 | 0.951759 | yes | calcium uniporter protein |
| Gb_12595 | -0.612783 | yes | voltage-dependent anion channel protein 2 |
| Gb_20078 | -0.475788 | yes | voltage-dependent anion channel protein 2 |
| Gb_41359 | -0.81959 | yes | voltage-dependent anion channel protein 2 |
| Gb_01086 | -5.71993 | yes | Calmodulin |
| Gb_03898 | -2.12156 | yes | Calmodulin |
| Gb_09202 | -1.49002 | yes | Calmodulin |
| Gb_11457 | 0.688852 | no | Calmodulin |
| Gb_11458 | 0.0711812 | no | Calmodulin |
| Gb_13855 | -1.87992 | yes | Calmodulin |
| Gb_13856 | -1.67598 | yes | Calmodulin |
| Gb_15095 | 0.394284 | no | Calmodulin |
| Gb_15575 | -2.35452 | yes | Calmodulin |
| Gb_16484 | -1.06703 | yes | Calmodulin |
| Gb_22573 | -1.97884 | yes | Calmodulin |
| Gb_28442 | -1.6166 | no | Calmodulin |
| Gb_30717 | 0.486202 | yes | Calmodulin |
| Gb_30819 | -1.03461 | yes | Calmodulin |
| Gb_35180 | -0.704134 | yes | Calmodulin |
| Gb_00926 | -0.00535254 | no | CDPK, calcium-dependent protein kinase |
| Gb_05867 | -0.242117 | no | CDPK, calcium-dependent protein kinase |
| Gb_06650 | 0.200202 | no | CDPK, calcium-dependent protein kinase |
| Gb_11259 | -1.57813 | yes | CDPK, calcium-dependent protein kinase |
| Gb_22471 | -0.0291192 | no | CDPK, calcium-dependent protein kinase |
| Gb_22778 | 0.42596 | yes | CDPK, calcium-dependent protein kinase |
| Gb_26648 | 0.5844 | yes | CDPK, calcium-dependent protein kinase |
| Gb_29448 | 0.0461335 | no | CDPK, calcium-dependent protein kinase |
| Gb_31292 | 0.205622 | no | CDPK, calcium-dependent protein kinase |
| Gb_37721 | -0.0926456 | no | CDPK, calcium-dependent protein kinase |
| Gb_38179 | 0.210168 | no | CDPK, calcium-dependent protein kinase |
